# Efficacy of Seed Priming With Cucurbitacin Phytonematicides Against *Meloidogyne incognita* on Pea

**DOI:** 10.3389/fmicb.2022.863808

**Published:** 2022-06-10

**Authors:** Phatu William Mashela, Kgabo Martha Pofu, Moshibudi Paulina Bopape-Mabapa

**Affiliations:** Department of Plant Production, Soil Science and Agricultural Engineering, Green Biotechnologies Research Centre of Excellence, University of Limpopo, Polokwane, South Africa

**Keywords:** ingredient, cucurbitacin A, cucurbitacin B, hypogeal germination, phytonematicide, nematode management, PAD technology

## Abstract

The cost and environment concerns of existing drench-application technologies of cucurbitacin phytonematicides resulted in conceptualization of the priming-and-drying (PAD) technology of seeds with hypogeal germination. The preliminary observations suggested that the PAD technology improved seed germination, plant growth, and vigor in pea (*Pisum sativa*), with limited information on suppression of root-knot (*Meloidogyne* species) nematodes. Post-soaking pea seeds in geometric concentrations of Nemarioc-AL and Nemafric-BL phytonematicides, seedlings were raised in greenhouse and on microplot experiments during 2019 and validated in 2020. At 60 days after inoculation with 300 eggs + second-stage juveniles (J2) of *M. incognita* seasonal data were pooled. Gall rating, eggs in root, and J2 in root vs. Nemarioc-AL phyto nematicide in greenhouse and on microplot exhibited negative quadratic relations, with models explained by 80–85% and 89–94% associations, respectively. Similarly, for the respective sites negative quadratic models for nematode variables vs. Nemafric-BL phytonematicide were explained by 82–93% and 90–94% associations, respectively. In conclusion, pea seed remnants belowground gradually released cucurbitacins into the rhizosphere throughout the growing period, thereby suppressing nematode population densities, and therefore, the PAD technology has the potential for assessment in a large-scale application of cucurbitacin phytonematicides for pea production.

## Introduction

Worldwide, root-knot (*Meloidogyne* species) nematodes remain a serious threat to food security, with limited management options. The southern root-knot (*Meloidogyne incognita* Kofoid and White, 1919) Chitwood nematode has internationally been viewed as being more aggressive than *M. javanica* (Treub) Chitwood (Taylor and Sasser, 1978). In South Africa, the 2 *Meloidogyne* species occur mostly as mixed populations, with *M. javanica* being more aggressive than *M. incognita* (Kleynhans et al., 1996). The 2 species each has races, defined as morphologically being similar, but with the distinct differential host plants. The existence of races makes the management of the genus using nematode resistance in crops difficult. Traditionally, nematode population densities were managed using the highly effective fumigant nematicides, which were applied as pre-plant products due to their phytotoxicity challenges. Additionally, the biocidal nature of fumigant nematicides, their extended persistence in the soil and overall environment-unfriendliness, resulted in international withdrawal of the products from the agrochemical markets. Similarly, their counterparts, the systemic nematostatic chemical products, due to limited information on their acropetal and basipetal movements, resulted in the substantial quantities of chemical residues in produce, with high consumer fatalities in various countries (Essumang et al., 2017). Notwithstanding the widespread use of chemicals, nematode challenges could not be completely eliminated due to existence of inherent intrinsic survival strategies in certain stages of nematodes (McSorley, 2003). Additionally, nematostatic effects on nematodes were notably reversible (Noling, 2019).

Before the 2005 withdrawal of fumigant nematicides and the scaling down of nematostatic products, the global annual crop yield losses induced by nematodes were estimated at US$126 billion (Chitwood, 2003). However, at 3 and 8 years post their withdrawal, the respective losses were at US$157 billion (Abad et al., 2008) and US$173 billion (Einhellig, 2013), translating to relative increases in crop losses of 25 and 37%, respectively. Although global crop losses due to *Meloidogyne* species were not documented prior to the withdrawal of fumigant nematicides in 2005, 3 years after the withdrawal global estimates amounted to US$157 billion (Abad et al., 2008). Ever since the withdrawal, several alternative nematode management strategies were developed, but with their unique inherent challenges. For example, the cucurbitacin phytonematicides, Nemarioc-AL, and Nemafric-BL phytonematicides were developed as such alternatives using effective microorganisms fermentation of dried fruits from wild cucumber [*Cucumis myriocarpus* (Naude.)] and wild watermelon (*Cucumis africanus* L.), respectively (Mashela et al., 2017). In either granular (Nemarioc-AG, Nemafric-BG) or liquid (Nemarioc-AL, Nemafric-BL) formulation, the products consistently suppressed population densities of *Meloidogyne* species in soil and root (Mashela, 2002; Mashela et al., 2011, 2015, 2017; Tseke et al., 2013; Sithole et al., 2016; Tseke and Mashela, 2017). The efficacies of the products on promoting plant growth and nematode suppression were comparable to those of aldicarb, fenamiphos, and Velum (Mashela et al., 2008; Seshweni, 2017).

Cucurbitacin phytonematicides in granular formulation were applied using the ground leaching technology (GLT) system, which comprises applying small quantities (1–5 g or 2%) of the product in a shallow circular furrow around the seedling at transplanting, and then covering with soil prior to leaching using irrigation water (Mashela, 2002). In contrast, in liquid formulation (L) the products were applied through the botinemagation application technology (BAT), which constitutes the application of botanicals through irrigation water (Mashela et al., 2020). Generally, GLT and BAT are suitable for use in smallholder and large-scale farming systems, respectively. Each technology has advantages and disadvantages, with the latter such as high costs and the discharge of large quantities of active ingredients into soil. Efforts had been underway to investigate alternative application technologies with the view of ameliorating the disadvantages of the 2 existing application technologies.

In plant nematodes, yield loss is directly proportional to the initial nematode population densities (Pi) that would infect the developing root system (Seinhorst, 1965). Thus, seed priming would be ideal for suppressing Pi as observed previously in seed dressing of cotton (*Gossypium hirsutum* L.), sunflower (*Helianthus annuus* L.), okra (*Abelmoschus esculentus* L.) Moenchand soybean [*Glycine max* (L.) Merr] with various active ingredients, primarily those of synthetic chemical nematicides such as abamectin (Monfort et al., 2006; De Almeida et al., 2017). Seed germination was previously shown to be highly sensitive to cucurbitacin phytonematicides, particularly when used as granular formulation (Martin and Blackburn, 2003; Mafeo and Mashela, 2009, 2010; Mazloom et al., 2009; Mafeo et al., 2011; Gautam et al., 2017). The challenge of phytotoxicity in cucurbitacin phytonematicides was eventually resolved by developing the Mean Concentration Stimulation Point (MCSP) from biological indices generated using the Curve-fitting Allelochemical Response Dose (CARD) algorithm computer model (Liu et al., 2003; Mashela et al., 2015). MCSP was described as the concentration of a phytonematicide that would not induce phytotoxicity to the test plants, but would consistently suppress nematode population densities of the test nematode species (Mashela et al., 2015).

On the basis of seedling emergence, plants have 2 seed categories, those with epigeal and hypogeal germination attributes. In epigeal germination, the seed remnants, which contain etiolated cotyledons, endosperm, and all seed layers, are pulled out of soil surface by the emerging seedlings. In contrast, in seeds with hypogeal germination, seed remnants remain belowground. Most seeds have epigeal germination, whereas pea (*Pisum sativa* L.), maize (*Zea mays* L.) and certain runner bean (*Phaseolus* species) cultivars have hypogeal germination attributes. Pea and maize are each highly susceptible to *Meloidogyne* species (Windham and Williams, 1987; Gautam et al., 2017). Specifically, this nematode genus is 1 of the major limiting factors in pea production, with yield losses ranging from as high as 33% to complete crop failure (Upadhyay and Dwivedi, 1987). The use of seeds with hypogeal germination, where seed remnants serve as carriers of active ingredients of cucurbitacin phytonematicides, was motivated by notable advances in seed priming-and-drying (PAD) technology, where 2 chemical phases of seed germination were identified (Lutts et al., 2016). In our preliminary trials using PAD technology with cucurbitacin phytonematicides, we observed that the technology eliminated phytotoxicity, without information on whether using the technology on seeds with hypogeal germination would manage nematode population densities. The objective of this study was to determine the effects of seed remnants of hypogeal pea seeds that were primed in solutions of Nemarioc-AL and Nemafric-BL phytonematicides on suppression of *M. incognita* population densities.

## Materials and Methods

### Growth Conditions

The study was conducted under greenhouse and microplot conditions at the Green Biotechnologies Research Centre of Excellence, University of Limpopo, South Africa (23°53′10″S, 29°44′15″E). Trials for each product were separately initiated in autumn February-April 2019 and validated in 2020. Due to the size of the greenhouse (100 m × 20 m), along with wind streams created by heat-extracting fans, conditions inside the facility were fairly heterogeneous. The greenhouse had ambient day/night temperatures averaging 28/18°C, with high temperatures controlled using thermostatically-activated fans on 1 end and a wet wall on the opposite end, with relative humidity ranging from 70 to 75%. The microplot trial was initiated on plots adjacent the greenhouse facility, with summer rainfall averaging 500 mm per annum and daily maximum temperature ranging from 28 to 38^°^C.

### Preparation of Experimental Sites

In the greenhouse, 20-cm-diameter plastic pots (2,700 ml soil) were placed on benches at 0.3 m × 0.25 m spacing, whereas artificial microplots were established using 30-cm-diameter plastic pots (10,000 ml soil) inserted in 20-cm-deep holes at 0.6 × 0.3 m spacing. In each trial, pots were filled with steam-pasteurized (300°C for 1 h) loamy soil.

### Preparation of Phytonematicides

Fresh fruits were collected from cultivated plots of *C. myriocarpus* and *C. africanus* for Nemarioc-AL and Nemafric-BL phytonematicides, respectively. Fruits were rinsed in chlorinated tap water, cut into pieces and dried at 52^°^C in air-forced oven. Dried material were ground in the Thomas Model 4 Wiley Mill and fermented using effective microorganisms (EM) (Higa and Parr, 1994). Briefly,40 g dried fruit of *C. myriocarpus* and 80 g dried of *C. africanus* were each fermented using South Africans strains of EM, comprising yeast, lactic acid bacteria, photosynthetic acid bacteria and actenomycete bacteria for 14 days, with pH of the products reduced from neutral to 3.7 (Mashela et al., 2017).

### Priming-and-Drying Technology

A total of 20 pea seeds cv. “Evergreen” were primed in 30 ml solutions of 0, 2, 4, 8, 16, 32, and 64% Nemarioc-AL or Nemafric-BL phytonematicide, with dilutions achieved using chlorine-free tap water. Primed seeds in different containers were placed in an air-forced oven at 25^°^C for 2 h to enhance imbibition and the initiation of Phase I germination processes (Lutts et al., 2016). Solutions were discarded, with seeds pressed between laboratory paper towel to remove excess solution, and then further dried in air-forced oven at 30^°^C for 12 h to suspend activities of Phase I germination process.

### Treatments and Experimental Design

Treatments were arranged in a randomized complete block design, with six replications in greenhouse and eight replications on microplot. In the greenhouse blocking was done for wind streams generated by heat-extracting fans and on microplots for shading by windbreak trees in the late afternoon.

### Inoculation With Nematodes

*Meloidogyne incognita*, originally derived from the Agricultural Research Council (Tropical and Subtropical Fruit) of South Africa for inoculation, was multiplied on greenhouse-raised nematode-susceptible tomato cv. “Floradade.” The inoculum was prepared by extracting eggs and J2 from roots in 1% NaOCl solution (Hussey and Barker, 1973). At 7 days after complete seedling emergence, seedlings were thinned to 1 per pot and each inoculated by dispensing ca. 300 eggs + J2 of *M. incognita* using a 20 ml plastic syringe into 5-cm-deep holes on the cardinal sides of each seedling at 5-cm away from the stem, with holes covered using the growing medium. The low inoculation was intended to prevent competition for infection sites.

### Cultural Practices

Each plant was irrigated every other day using a 500 ml container of chlorine-free tap water for the first 30 days after sowing; thereafter a 600 ml container was used in the greenhouse and on microplots, respectively. At 5 leaf-stage, seedlings were fertilized with 5 g N:P:K 2:3:2 (22) per plant, which provided a total of 155 mg N, 105 mg P, and 130 mg K per ml of water and 1 g N:P:K 2:1:2 (43) per plant to provide a total of 0.175 mg N, 0.16 mg K, and 0.16 mg P, 0.45 mg, 0.378 mg Fe, 0.0375 mg Cu, 0.175 mg Zn, 0.5 mg B, 1.5 mg Mn, and 0.035 mg Mo per ml chlorine-free tapwater. Daily monitoring and scouting for greenhouse whiteflies (*Trialeurodes vaporariorum* Westwood) was performed, with plants sprayed once using Whitefly insecticide at 5 ml/10 L water when insect population densities were beyond 100 entities per trial.

### Data Collection

At 60 days after sowing (allowing 2 nematode life cycles), plant tops were severed and roots removed from pots, with roots immersed in water and slightly shaken to dislodge off soil particles without detaching egg masses. Moist roots were blotted dry in a laboratory paper towel and weighed. Root galls were assessed using the 0–5 gall rating scale (Hussey and Barker, 1973). Eggs + J2 were extracted from total root system using the modified maceration and blending method for 30 s in 1% NaOCl solution (Taylor and Sasser, 1978; Marais et al., 2017). Nematodes in soil samples were not assessed. Eggs + J2 from roots were counted from 5 ml aliquot using a 60 X magnification under a stereo microscope Stemi 508 (Zeiss, Jena, Germany). Except for root galling, nematode variables were expressed per g freshroot.

### Data Analysis

Except for root galling, prior to analysis all nematode data were transformed using log_10_(x + 1). In all datasets, prior to analysis the geometric series (x-axis) was expressed as exponential series [2^0^, 2^1^, 2^2^, 2^3^, 2^4^, 2^5^, and 2^6^], with the series log-transformed using log_2_2*^x^* = x to generate equidistant integers [0, 1, 2, 3, 4, 5, 6, and 7%] in order to enhance normality (Causton, 1977). Seasonal interactions were assessed for each dataset using Statistix 10.0 software. Since the seasonal interaction for each variable was not significant (*p* ≥ 0.05), data were pooled for greenhouse (*n* = 84) and microplot (*n* = 112) trials. The Shapiro-Wilk test was performed to determine the normality of the datasets (Shapiro and Wilk, 1965; Ghasemi and Zahediasl, 2012). Since the datasets exhibited normal distribution, they were subjected to analysis of variance, with significant treatment means further subjected to lines of the best fit *post-hoc* test of mean separation using the Tukey test. Unless stated otherwise, data were described at the probability level of 5%.

## Results

### Response of Root Galling to Phytonematicides in Priming-and-Drying Technology

**FIGURE 1:**
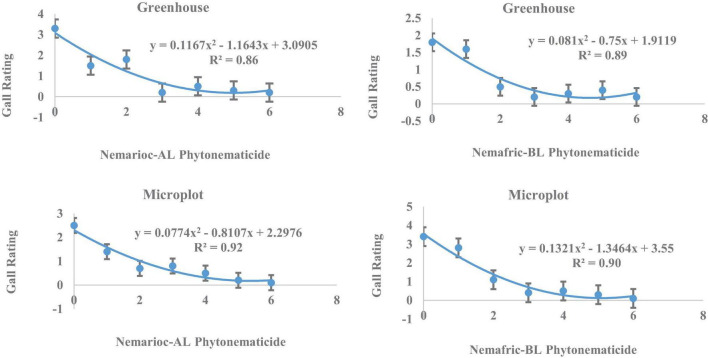
Response of *M. incognita* root galls in pea seeds primed in cucurbitacin phytonematicides from root of plants raised in greenhouse and microplot conditions (Bar represents mean ± standard error) (The x-axis comprised transformed data, where 0 = 0, 1 = 2, 2 = 4, 3 = 8, 4 = 16, 5 = 32, and 6 = 64% phytonematicide).

In greenhouse and on microplot trials, treatment effects were highly significant on all nematode variables measured (data not shown), with mean separation demonstrating clear patterns of differences on untreated controls and phytonematicides (Table 1). Under both environments, gall rating vs. Nemarioc-AL phytonematicide exhibited negative quadratic relations, with the models explained by 86 and 92%, respectively (Figure 1). Using x = b_1_/2b_2_ relation from the quadratic equation Y = b_2_x^2^ + bx + c for root galling of plants in the greenhouse and microplot trials, the minima for galling were achieved at 4.99 and 5.237% Nemarioc-AL phytonematicide, respectively. Similarly, models for root galling vs. Nemafric-BL phytonematicide at the respective sites were explained by 89 and 90% associations (Figure 1), with the minima achieved at 4.630 and 5.096% Nemafric-BL phytonematicide.

**TABLE 1:** Effects of priming pea seeds in Nemarioc-AL and Nemafric-BL phytonematicides on pea plants subjected to *M. incognita*.

	Greenhouse		Microplot	
Concentration (%)	Nemarioc-	Nemafric-	Nemarioc-	Nemafric-
**Root gall (total roots)**				
0 (0)	3.3^az^	1.8^a^	2.5^a^	3.4^a^
1 (2)	1.5^b^	1.6^a^	1.4^b^	2.8^b^
2 (4)	0.8^c^	0.5^b^	0.7^c^	1.1^c^
3 (8)	0.2^c^	0.2^b^	0.8^c^	0.4^c^
4 (16)	0.5^c^	0.3^b^	0.5^c^	0.5^c^
5 (32)	0.3^c^	0.4^b^	0.2^c^	0.3^c^
6 (64)	0.2^c^	0.2^b^	0.1^c^	0.1^c^
**Eggs/g fresh root**				
0 (0)	1.8^a^	2.5^a^	3.1^a^	2.8^a^
1 (2)	0.8^b^	1.1^b^	1.9^b^	2.3^a^
2 (4)	0.6^b^	1.6^b^	1.8^b^	0.8^b^
3 (8)	0.5^b^	0.3^c^	0.4^c^	0.5^b^
4 (16)	0.6^b^	0.5^c^	0.2^c^	0.6^b^
5 (32)	0.1^b^	0.4^c^	0.3^c^	0.5^b^
6 (64)	0.4^b^	0.3^c^	0.5^c^	0.2^b^
**Second-stage juveniles/g fresh root**				
0 (0)	3.5^a^	3.1^a^	3.5^a^	2.2^a^
1 (2)	1.8^b^	1.8^b^	2.4^b^	1.9^ab^
2 (4)	1.2^b^	1.4^b^	2.3^b^	1.3^b^
3 (8)	1.4^b^	1.2^b^	1.4^c^	0.5^c^
4 (16)	1.0^bc^	1.0^bc^	1.8^c^	0.6^c^
5 (32)	0.2^c^	0.7^c^	1.5^c^	0.1^c^
6 (64)	0.3^c^	0.5^c^	1.1^c^	0.3^c^

*^z^Column means followed by the same letter were not different (p ≤ 0.05) according to Tukey test.*

### Response of Eggs in Root to Phytonematicides in Priming-and-Drying Technology

**FIGURE 2:**
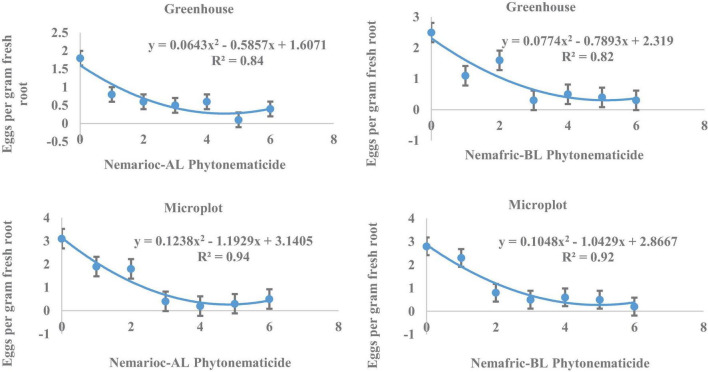
Response of *M. incognita* eggs in root of pea seeds primed in cucurbitacin phytonematicides and seedlings raised in greenhouse and microplot conditions (Bar represents mean ± standard error) (The x-axis comprised transformed data, where 0 = 0%, 1 = 2%, 2 = 4%, 3 = 8%, 4 = 16%, 5 = 32%, and 6 = 64% phytonematicide).

At the 2 sites, eggs in root vs. Nemarioc-AL phytonematicide exhibited negative quadratic relations, with the models explained by 84 and 94% associations, respectively (Figure 2). The minima for eggs in root were achieved at 4.554 and 4.818% Nemarioc-AL phytonematicide, respectively. Models for eggs in root vs. Nemafric-BL phytonematicide were explained by 82 and 92% associations, respectively, with the respective minima achieved at 5.099 and 4.976% Nemafric-BL phytonematicide.

### Response of Second-Stage Juveniles in Root to Phytonematicides in Priming-and-Drying Technology

**FIGURE 3:**
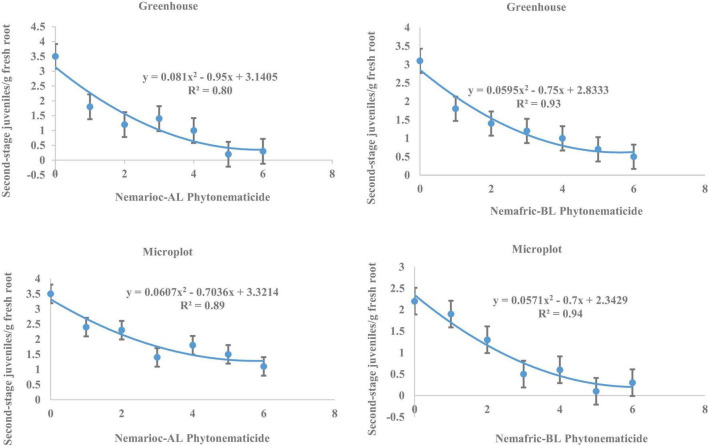
Response of *M. incognita* second-stage juveniles in roots of pea seeds primed in cucurbitacin phytonematicides and seedlings raised in greenhouse and microplot conditions (Bar represents mean ± standard error) (The x-axis comprised transformed data, where 0 = 0%, 1 = 2%, 2 = 4%, 3 = 8%, 4 = 16%, 5 = 32%, and 6 = 64% phytonematicide).

At the 2 sites, J2 in root vs. Nemarioc-AL phytonematicide exhibited negative quadratic relations, with models explained by 80 and 89% associations, respectively (Figure 3). The respective minima for J2 in root were achieved at 5.864 and 5.796% Nemarioc-AL phytonematicide. Models for J2 in root vs. Nemafric-BL phytonematicide were explained by 93 and 94% associations, respectively, with the minima achieved at 6.303 and 6.130% Nemafric-BL phytonematicide.

## Discussion

The observations confirmed that priming pre-sown seeds of pea in cucurbitacin phytonematicides resulted in remnants being carriers of cucurbitacin active ingredients. The observed relationships were consistent with those in GLT and BAT systems, where cucurbitacin phytonematicides starting from 2% consistently suppressed nematode population densities of *Meloidogyne* species (Pelinganga and Mashela, 2012; Mashela et al., 2015, 2017). Results suggested that cucurbitacins in water were taken into the seed during the imbibition process. Generally, in most seeds the micropylar and chalazal canals provide specialized functions that enhance permeability of materials through the testa, with the underlying hypodermis, sclerenchyma, aerenchyma, and chlorenchyma layers having extensive intercellular spaces that serve the same purpose (Maila, 2015). The canals and intercellular spaces facilitate the movement of water to the embryo through the process of imbibition. Since after priming, seeds were dried, such canals and intercellular spaces could serve as reservoirs of cucurbitacins, which remained locked in seed remnants after all chemical bioactivities for embryo growth and germination have been accomplished. Apparently, when subjected to the PAD technology, cucurbitacins in pea seeds did not interfere with embryo growth and subsequent development of seedlings.

Suppression of nematodes in root served as indicator that the cucurbitacin reservoirs in the seed remnants were gradually released into the rhizosphere. Although it was not clear how long the seed remnants would continue to release cucurbitacins into the rhizosphere, in the current pea study the activity occurred as long as 60 days after inoculation with nematodes. The latter was substantially longer than the observed effects of synthetic chemical nematicides in epigeal seeds, where efficacy on nematode suppression lasted for no more than 15 days after sowing (Monfort et al., 2006; De Almeida et al., 2017). In our study, *M. incognita* was managed by the released cucurbitacins from seed remnants for at least 3 nematode life cycles, which was similar to situations where cucurbitacin phytonematicides were applied using GLT system (Mashela, 2002). Apparently, once cucurbitacins were in the canals and airspaces of seeds, temporary drying trapped the material inside the seed, but without interference with germination, which is also a chemical process. Once germination is completed by the radicle rapturing the testa at the micropylar end, the growing radicle is strategically positioned beneath the seed remnant. Cucurbitacins in seed remnants are gradually leached by irrigation water into the rhizosphere as radicle and seedling growth proceed. Suppression of nematode population densities through the priming technology as observed in our study was important since yield loss due to plant nematodes is directly proportional to the initial nematode population densities (Pi) that attack the developing lateral root system (Seinhorst, 1965). Additionally, it is important to note that the protective effects were extended to over 3 life cycles of *M. incognita*.

Model associations for nematode variables vs. Nemafric-BL phytonematicide were more or less similar to those of Nemarioc-AL phytonematicide, regardless of the experimental site. The equivalence of the associations did not confirm many other observations under GLT and BAT, where effects of the 2 products on nematode suppression significantly differed (Pelinganga and Mashela, 2012; Dube, 2016; Mashela et al., 2017). The observed differences had previously been explained on the basis of the chemistry of cucurbitacin A (C_32_H_46_O_9_), which is slightly polar, but with limited solubility in water (Jeffrey, 1980). The molecule rapidly breaks down into cucumin (C_36_H_46_O_9_) and leptodermin (C_36_H_46_O_8_) chemical molecules (Chen et al., 2005). In contrast, cucurbitacin B (C_32_H_46_O_8_) is non-polar and insoluble in water, with the compound being highly stable (Jeffrey, 1980). All listed attributes could, to a certain degree, provide an explanation to some of the differences observed in nematode responses to the 2 products. The results suggested that cucurbitacins in seed remnants of pea remained stable as opposed to when dispersed in soil solutions, where bioremediation factors such as ecdozoans, such as nematodes, have substantial effects on concentration of cucurbitacins (Mashela et al., 2022).

The minimum values of phytonematicides observed in the current study might not be of practical application in pea production. Ordinarily, a non-phytotoxic concentration for pea plants should still be empirically determined using the CARD algorithm model (Mashela et al., 2017). The empirically based non-phytotoxic concentration would be the ideal 1 for use during priming pea seeds in PAD technology instead of using the minimum values that were derived in the current study.

## Conclusion

The remnants of pea seeds with hypogeal germination attributes successfully served as carriers of active ingredients of cucurbitacin phytonematicides in priming technology intended to manage population densities of *Meloidogyne* species. Under both greenhouse and microplot conditions, regardless of the phytonematicide used in seed priming, PAD technology consistently suppressed population densities of *Meloidogyne* species. In future, the technology would be extended to other seeds with hypogeal germination attributes such as maize under both irrigation and dry land farming systems prior to extension of such crops under large-scale farming systems. Additionally, the development of MCSP for seeds under PAD technology, along with investigation related to the persistence of cucurbitacins in seed remnants, are some of the areas of interest for future commercialization of the technology.

## Data Availability Statement

The original contributions presented in this study are included in the article/supplementary material, further inquiries can be directed to the corresponding author.

## Author Contributions

PM conceptualized the study with all authors. All authors contributed equally to conducted of the experiments, data analysis, and discussion.

## Conflict of Interest

The authors declare that the research was conducted in the absence of any commercial or financial relationships that could be construed as a potential conflict of interest.

## Publisher’s Note

All claims expressed in this article are solely those of the authors and do not necessarily represent those of their affiliated organizations, or those of the publisher, the editors and the reviewers. Any product that may be evaluated in this article, or claim that may be made by its manufacturer, is not guaranteed or endorsed by the publisher.
